# Distribution of coastal blue carbon habitats in Sweden and their exposure to anthropogenic pressure

**DOI:** 10.1007/s13280-025-02290-x

**Published:** 2025-12-03

**Authors:** Sara Braun, Martin Dahl, Maria E. Asplund, Karin Ebert, Mats Björk, Martin Gullström

**Affiliations:** 1 School of Natural Science, Technology and Environmental Studies, Södertörn University, 141 86 Huddinge, Sweden; 2 Department of Biological and Environmental Sciences, University of Gothenburg, Kristineberg, 451 78 Fiskebäckskil, Sweden; 3 Department of Ecology, Environment and Plant Sciences (DEEP), Stockholm University, 106 91 Stockholm, Sweden

**Keywords:** Coastal management, Emergent blue carbon ecosystems, Habitat mapping, Landscape analysis, Vegetated coastal habitats

## Abstract

Understanding where blue carbon habitats occur and how they are affected by human activity contributes to effective management of natural carbon sinks. Here, we compiled geographical data for Sweden to map the distribution of coastal vegetated blue carbon (BC) habitats. The mapping effort focused on well-recognised (salt marshes and seagrass meadows) and emergent BC habitats (other rooted submerged macrophytes and forested wetlands). We also estimated the exposure to anthropogenic pressures on coastal BC habitats based on their proximity to land-based human activities, and subsequently, the portion of these BC habitats that were located within protected areas. The total area of BC habitats was estimated to around 1850 km^2^, corresponding to ca. 35% of the Swedish coast. Seagrass meadows and other rooted submerged macrophytes were dominating, covering about 1500 km^2^. Around 22% of the mapped BC habitats were expected to be exposed to high pressures from land-based human activities due to their location, while BC habitats within protected areas were often less exposed. This nationwide assessment of coastal vegetated BC habitats accentuates the need for strengthening conservation prioritisation to maximise the carbon storage potential of BC habitats.

**Supplementary Information:**

The online version contains supplementary material available at 10.1007/s13280-025-02290-x.

## Introduction

Mapping coastal habitats is fundamental for understanding their spatial distribution and structural configuration. The spatial arrangement of these habitats has profound implications for biodiversity, population dynamics and essential ecosystem functions, including shoreline stabilisation, nutrient cycling and blue carbon (BC) sequestration (Gracia et al. 2018; Gullström et al. 2018; Loke et al. 2019). Blue carbon refers to the natural carbon sinks of the ocean (Nellemann 2009), and BC research in temperate regions has mainly focused on seagrass meadows, salt marshes and open wetlands due to their high carbon sequestration capacity (Drake et al. 2015; Röhr et al. 2018; Prentice et al. 2020). These widely distributed vegetated habitats efficiently sequester CO_2_ via photosynthesis and through trapping and accumulation of allochthonous organic carbon (OC) from the surrounding environment, which is mainly stored in the underlying sediments (Howard et al. 2014). There are also other emergent vegetated coastal habitats with similar traits as seagrass meadows, salt marshes and open wetlands that have the potential to be included among the recognised coastal BC habitats (Howard et al. 2023). Recent studies have highlighted several coastal habitats, such as macroalgae beds (Krause-Jensen et al. 2018; Raven 2018), mudflats (Epstein et al. 2025), unvegetated sediments (Wikström et al. 2025), rhodolith beds (James et al. 2024), rooted macrophytes other than seagrass (Hillmann et al. 2020) and tidal freshwater forests (Adame et al. 2024), which may all have the potential to be considered BC habitats and important natural carbon sinks. Such an expansion of contemporary BC habitats in the coastal zone would be beneficial for efficient coastal management of natural OC sinks and could contribute to the overall climate mitigation capacity of coastal areas.

**Fig. 1:**
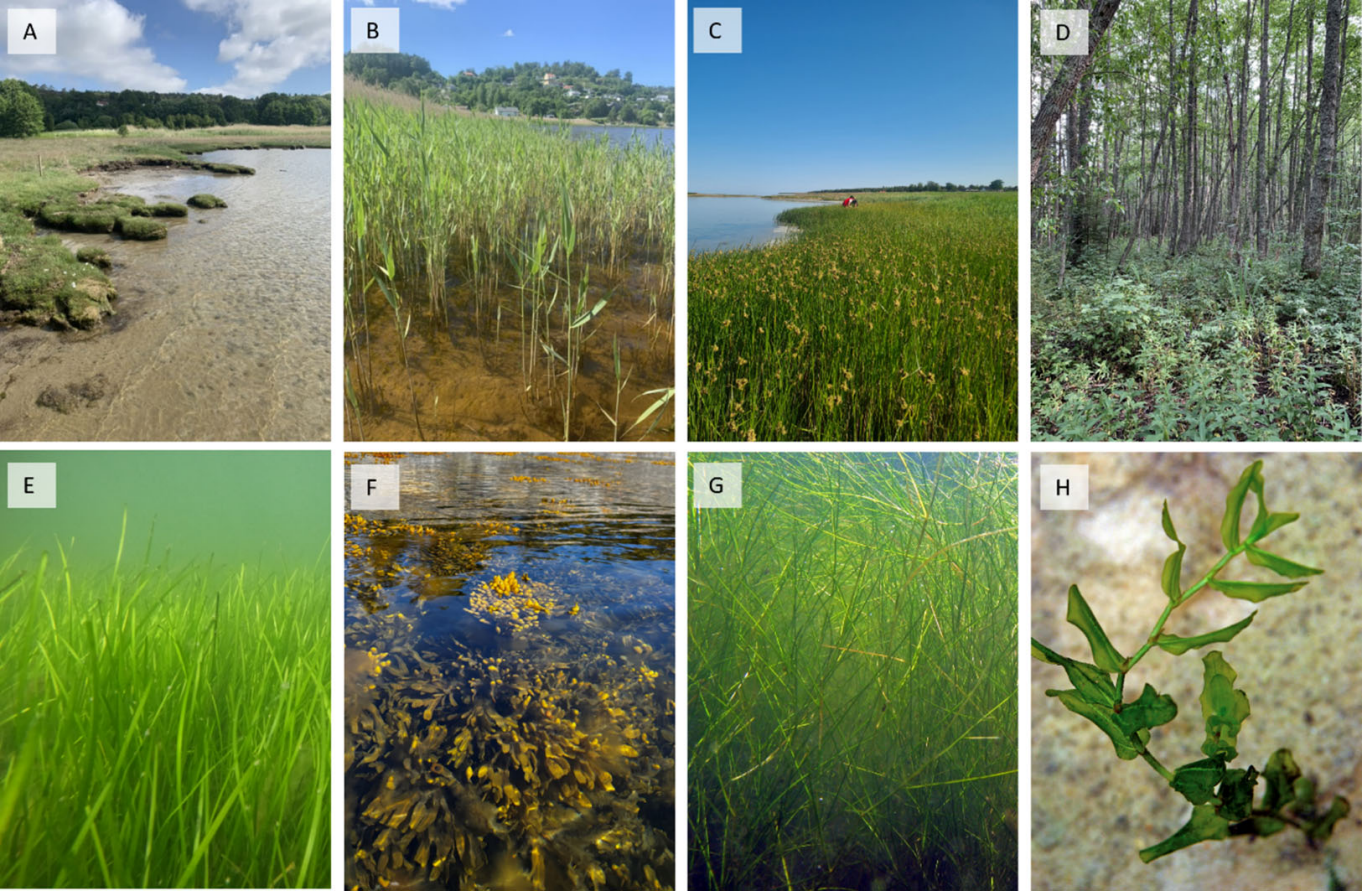
Coastal vegetated habitats with the potential for BC storage in the study area: **A** open wetland, **B** reed (*Phragmites australis*) bed, **C** bullrush (*Bolboschoenus maritimus*) marsh, **D** coastal forested wetland, **E** seagrass (*Zostera marina*) meadow, **F** macroalgae (*Fucus vesiculosus*) and **G** and **H** other rooted macrophytes (e.g. in this figure, *Stuckenia pectinata* and *Potamogeton perfoliatus*). Photos A–F by Sara Braun, Sara C. Forsberg and Maria E. Asplund. Photos G and H by Adrian Jones, Integration and Application Network (ian.umces.edu/media-library)

In cold-temperate Sweden, coastal vegetated habitats with potential for BC storage (including both recognised and emergent habitats) comprise seagrass meadows and other rooted submerged macrophytes, semi-submerged vegetated areas (e.g. saltmarshes, reed and bullrush habitats), coastal forested wetlands and macroalgae via their indirect contribution (Table 1 and Fig. 1). Seagrasses and other rooted submerged macrophytes are widely found on soft-bottom sediments in shallow coastal areas (Hemminga and Duarte 2000; Hillmann et al. 2020). The dominating seagrass species in Sweden, *Zostera marina* (eelgrass), has a distribution range from the near-oceanic waters of Skagerrak to the northern Baltic Proper where the low salinity (~ 5) constitutes a limit for seagrass growth (Boström et al. 2014). In the brackish Baltic Sea, it is also common to find other types of rooted macrophytes, such as *Ruppia* spp., *Potamogeton* spp., *Myriophyllum* spp. and *Zanichellia* spp. (Kautsky 1988; Pitkänen et al. 2013). The common reed (*Phragmites australis*), one of the world’s most productive and widespread plant species (Clevering and Lissner 1999), and bullrush beds, which are usually dominated by the flowering plant species *Bolboschoenus maritimus* and *Schoenoplectus tabernaemontani*, are often found along the Baltic Sea coast (Gunnarsson 2009; Altartouri et al. 2014). Coastal forested wetlands are usually a mix of coniferous and deciduous tree species, although frequently dominated by *Alnus glutinosa*. Generally, there is a lack of studies evaluating the OC storage in reed and bullrush beds, coastal forested wetlands and habitats dominated by rooted submerged macrophytes other than seagrasses. Recent studies, however, show that carbon stocks in these habitats range between below 2 and almost 90 kg OC m^−2^ (at 1 m sediment depth), which is similar or even higher levels compared to stocks in the well-recognised BC habitats (i.e. seagrass meadows and saltmarshes) (Hillmann et al. 2020; Buczko et al. 2022; Graversen et al. 2022; Adame et al. 2024; Leiva‐Dueñas et al. 2024).

**Table 1:** Vegetated habitats with potential for BC storage in the study area based on the IPCC definition of BC habitats, which includes criteria for net CO_2_ uptake, long-term storage of OC and amenable management actions for avoided emissions/enhanced OC sequestration. *HELCOM definition (i.e. Kattegat, Danish Straits, Baltic Proper, Bothnian Sea, Bothnian Bay, Gulf of Riga and Gulf of Finland), **open wetland and reed/bullrush was merged in the mapping, ***results from outside the study area, which likely apply within the Baltic Sea and Skagerrak area as well

Well-recognised and emergent coastal vegetated Baltic Sea area* and Skagerrak blue carbon habitats				
Habitat	Net CO_2_ uptake	Long-term OC sinks	Amenable to management for avoided emission and/or enhanced sequestration	References
Seagrass	Yes	Yes	Yes	Jankowska et al. (2016), Moksnes et al. (2021), Asplund et al. (2022), Kindeberg et al. (2023), Leiva‐Dueñas et al. (2023), Dahl et al. (2024), Henriksson et al. (2024)
Open wetland**	Yes***	Yes	Yes***	Burden et al. (2013), Elschot et al. (2015), Witte and Giani (2016), Graversen et al. (2022), Leiva‐Dueñas et al. (2024)
Macroalgae	Yes	Potential transport of OM to adjacent habitats and deep ocean	Potential transport of OM to adjacent habitats and deep ocean	Krause-Jensen et al. (2018), Roth et al. (2023)
Reed/Bullrush**	Yes, long-term	Yes	Yes, likely	Brix et al. (2001), Leiva‐Dueñas et al. (2024)
Other rooted submerged macrophytes	Yes	Yes	Yes***	Hillmann et al. (2020), Roth et al. (2023), Wikström et al. (2025), Gubri et al. (2025)
Forested wetland	Yes***	Yes***	Yes***	Adame et al. (2024)

Human-caused stressors, such as urban development, eutrophication, overfishing and pollution, put pressure on the structure and functions of natural ecosystems and have led to large losses of coastal marine vegetated habitats during the last decades (Dunic et al. 2021; Murray et al. 2022). On the Swedish west coast, about 60% of the seagrass area were lost between 1980 and 2000s (still without any major recover), primarily due to coastal eutrophication (Baden et al. 2003; Nyqvist et al. 2009) and cascading effects from overexploitation of large predatory fish (Moksnes et al. 2008). Furthermore, changes in vascular- and charophyte plant distribution and species composition due to eutrophication were observed in the western part of the Gulf of Finland between the 1930s/1940s and early 2000s (Pitkänen et al. 2013). Over the past century, one-fourth of all wetlands in Sweden have been lost due to drainage aimed at improving agricultural and forestry conditions (Naturvårdsverket 2012). In terms of coastal wetlands, some areas have suffered from severe regressions following agricultural and coastal expansion (Gunnarsson 2009), and overgrowth by taller grasses such as reed as a response to the decline of traditional grazing and mowing in these environments (Dijkema 1990; Burnside et al. 2007). Current knowledge regarding the threat level and potential decline of coastal forested wetland habitats is limited.

Effective coastal management and conservation prioritisation require wide and in-depth habitat mapping efforts to accurately assess the distribution, coverage and the status of coastal habitats. Today, coastal managers commonly use a variety of analysis tools in order to identify, assess and mitigate threats to coastal habitats (Cruz-Ramírez et al. 2024). Generally, there is a lack of coastal habitat maps in many regions around the world due to limited geographical information, gaps in monitoring data, environmental changes and technological constraints, which hinders successful habitat conservation, resource management and climate adaptation measures (McKenzie et al. 2020; Macreadie et al. 2021). In Sweden, habitat mapping and monitoring is carried out using varying methods and involves multiple actors. For example, the national mapping and monitoring is carried out within the program NILS (“National Inventory of the Landscape in Sweden”; Inghe 2001) and the Swedish national land cover database provides a national map of habitats on land (Naturvårdsverket 2024). Within protected areas, mapping and monitoring are carried out within the scope of the EU Habitats Directive. For semi-submerged habitats (e.g. reed and bullrush beds) and especially for fully submerged habitats (e.g. seagrass meadows), mapping and monitoring at nationwide scale is more restricted.

In this study, we compiled existing data, which is based on remote sensing, spatial statistical modelling and ground-truth surveys, to create a comprehensive map covering the distribution of well-recognised (i.e. saltmarshes and seagrass meadows) and emergent vegetated BC habitats (including other rooted submerged macrophytes besides seagrass and coastal forested wetlands) along the Swedish coastline. To estimate the exposure to anthropogenic pressure on coastal BC habitats, we calculated an index based on landscape metrics in terms of the proximity to and total area of agricultural and urban areas in Sweden’s coastal drainage basins. Further, we assessed the spatial overlap of BC habitats and protected areas, as well as the level of pressure on BC habitats (using their proximity to land-based human activities as a proxy) within and outside protected areas.

## MATERIALS AND Methods

### Study area

**Fig. 2:**
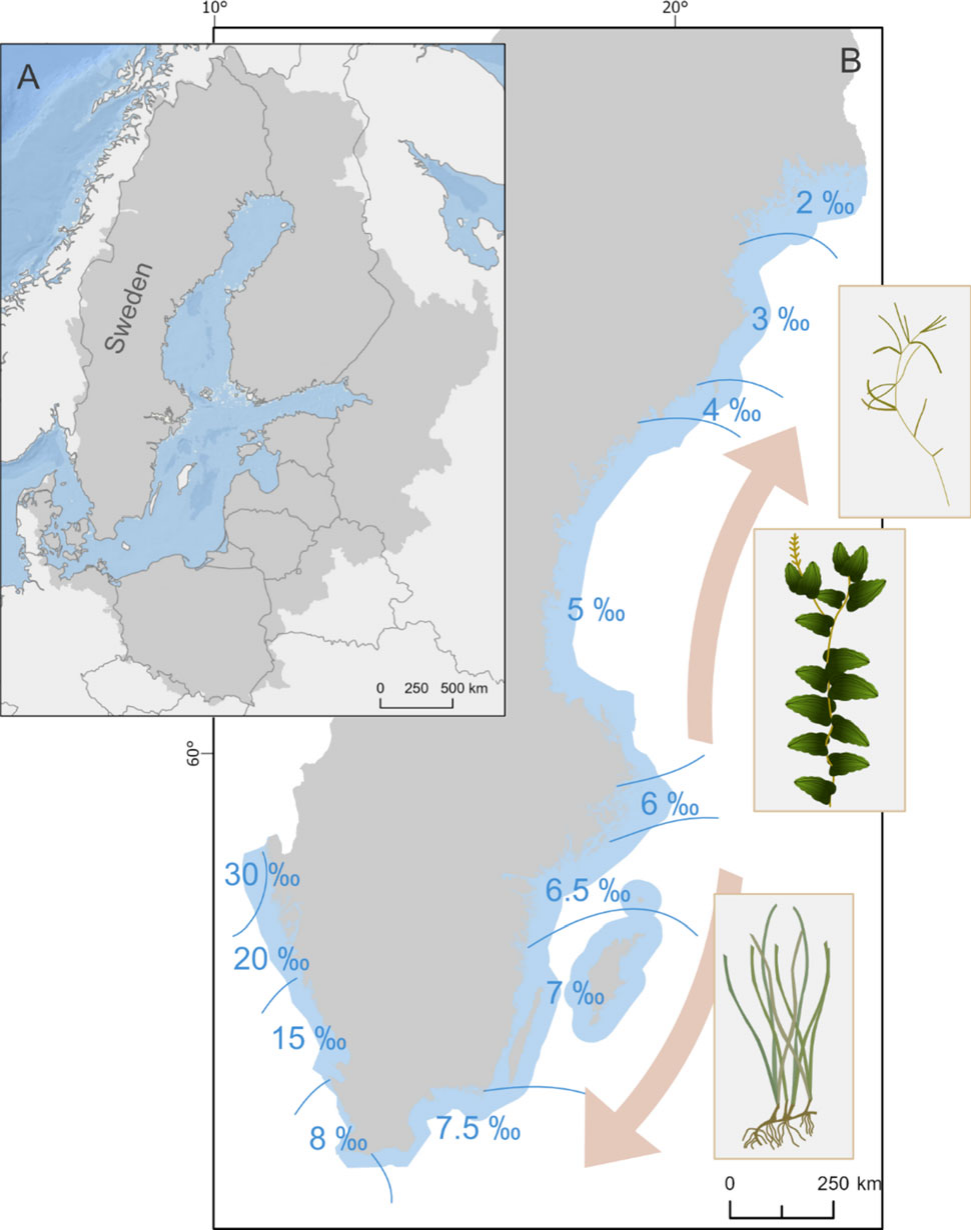
Catchment area (shown in grey) of the Baltic Sea area (**A**) and the salinity gradient (ranging between 2 and 8 in the Baltic Sea and between 8 and 30 on the Swedish west coast) and common rooted submerged macrophytes (from top to bottom: *Zanichellia* spp., *Potamogeton perfoliatus* and *Zostera marina*) along the Swedish coastline (**B**). Map sources: Baltic Sea catchment area from HELCOM map and data service, 2024 (HELCOM MADS; https://maps.helcom.fi/website/mapservice/) and background map freely available from the Swedish Mapping, Cadastral and Land Registration Authority

The coast of Sweden stretches from Skagerrak in the north-west, via Kattegat and the Belt Sea, to the Baltic Sea in the east (Fig. 2.). The Baltic Sea is a brackish inland water body connected with the main Atlantic Ocean via the Danish Straits (Leppäranta and Myrberg 2009). Kattegat and the Belt Sea are a mixture of brackish and oceanic water and Skagerrak is near-oceanic (Leppäranta and Myrberg 2009). The Baltic Sea is one of the most polluted seas in the world (HELCOM 2010), largely due to its large drainage area compared to its water volume and the long retention time of the water. The Swedish coast is severely impacted by land-based activities in its drainage basin, with increased pollution and nutrient loads since the last decades, which is especially pronounced in the Baltic Proper (Snoeijs-Leijonmalm et al. 2017). In Kattegat and Skagerrak, high sedimentation loads and recent coastal exploitation (e.g. building of harbours and jetties) have additionally led to large changes in coastal ecosystems (Eriander et al. 2017).

### Input data for the blue carbon habitat distribution map

**Fig. 3:**
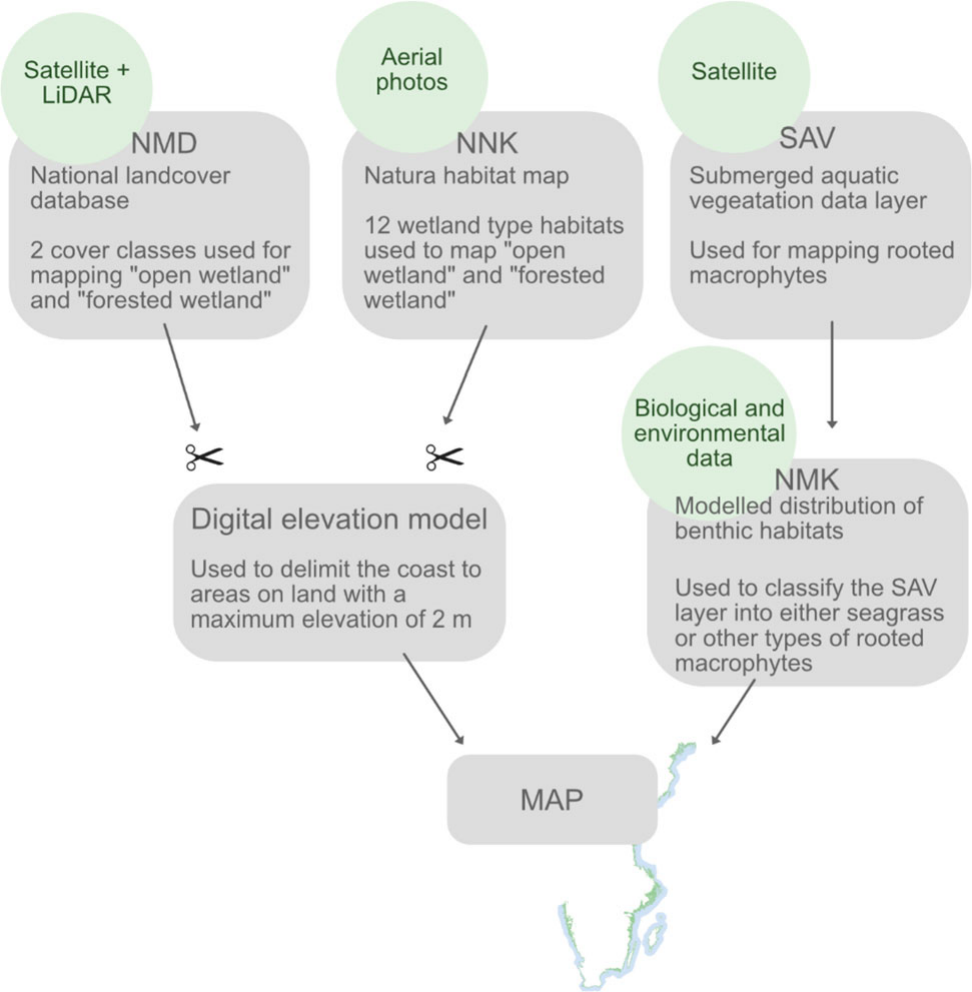
Overview of the input data compiled to map the distribution of well-recognised and emergent BC habitats along the Swedish coastline. The Swedish national land cover database (NMD) (Naturvårdsverket 2024) and the natura habitat map (NNK) (Naturvårdsverket 2023) were used to map “open wetland” and “forested wetland”. A national data layer of submerged aquatic vegetation (SAV) (Huber et al. 2022; Thomasdotter et al. 2024) and modelled distribution data of benthic habitats (NMK) (Carlström et al. 2010; Nyström Sandman et al. 2013) were used to map the distribution of seagrass and other rooted submerged macrophytes

The data compiled to produce a map of the distribution of recognised and emergent BC habitats along the Swedish coastline were based on available data layers from (1) the Swedish national land cover database (in Swedish: nationella marktäckedata, NMD) (Naturvårdsverket 2024), (2) the natura habitat map (in Swedish: Natura naturtypskartan, NNK) (Naturvårdsverket 2023), (3) a national database of submerged aquatic vegetation (SAV) (restricted to areas dominated by soft bottom) (Huber et al. 2022; Thomasdotter et al. 2024), and (4) modelled distribution data of benthic habitats (Carlström et al. 2010; Nyström Sandman et al. 2013) (Fig. 3).

The NMD contains land cover and land use data divided into 16 classes covering Sweden, which are mapped by integrating satellite data from Sentinel-2 and LiDAR (light detection and ranging) with 10 m spatial resolution. For the BC habitat distribution map, land cover classes corresponding to “open wetland” and “forest on wetland” were selected (see Table S1 in Supplementary Information for further details) and further processed in GIS software (ArcGIS Pro3.0). These two land classes were mapped with an accuracy of 80–100% and 70–80%, respectively. In contrast to the NMD raster layer, the NNK is a vector database, which includes the distribution of Natura 2000 habitat types in the EUs Habitats Directive, Annex 1 (EEG 92/443), as well as a selection of other habitat types (e.g. beach habitats along the Baltic coast not classified as habitat type classes 1220 or 1640 and cultivated open grasslands) within protected areas of Sweden. The NNK is based on the maps produced in a previous project (“Baseline Inventory of Natura 2000 and Protected Areas”; Naturvårdsverket 2009). When new areas are protected or added to Natura 2000, the Swedish Environmental Protection Agency carries out baseline mapping and the County Administrative Boards are responsible for quality control and keeping the data up-to-date (Naturvårdsverket 2023). From the NNK data layer, we combined a total of 13 wetland habitat types to map the distribution of “open wetland" and “forested wetland” (see a complete list in Table S2). The national SAV raster data layer is based on satellite data (10 m spatial resolution) for vegetation distribution in shallow soft-bottom areas for the years 2018–2022 (Huber et al. 2022; Thomasdotter et al. 2024). The SAV layer has a maximal depth limit of approximately 6 m. However, depending on the water quality and seabed reflectance, the depth limit can vary substantially along the coast and therefore most of the mapped SAV is within areas shallower than 3 m (Huber et al. 2022). The total accuracy of the SAV layer, mainly estimated from shallow (< 6 m) and sheltered areas, was nationally estimated to 72% with some variation across the different basins (Bothnian Bay: 69%, Baltic Proper: 77%, Skagerrak/Kattegat/Öresund area: 71%; Thomasdotter et al. 2024). Further, from using drone-generated ground-truth points about 30–65% (30% for the north-east coast, 65% for the south-east coast and 50% for the north-west coast) of the vegetation was visible using remote sensing (mainly deeper SAV areas were missed in the satellite images; Huber et al. 2022). The SAV data layer does not distinguish between different types of SAV, and therefore, in our mapping, we differentiated between SAV areas dominated by *Z. marina* and SAV areas dominated by other types of rooted macrophytes based on previous species distribution models of *Z. marina* (Carlström et al. 2010; Nyström Sandman et al. 2013). Detailed maps (10 m spatial resolution) of modelled *Z. marina* distribution were available for a few counties. Here, the modelled *Z. marina* distribution in the Södermanland and Östergötland counties was used. Areas with an overlap of the modelled potential distribution of *Z. marina* and SAV coverage according to the SAV data layer were classified as seagrass, while areas (solely within the extent of the SAV data layer) with no overlap were classified as other types of rooted macrophytes. For counties north of Södermanland, all SAV was classified as other types of rooted macrophytes besides seagrass, and for all counties south of Östergötland and on the west coast, all SAV was classified as seagrass. It should be noted that *Z. marina* and other types of rooted macrophytes  can occur in mixed meadows. Although *Z. marina* is the main type of rooted macrophyte in counties south of Östergötland, other types of rooted vegetation are also present in these areas. Likewise, seagrass meadows can be found north of Södermanland (up to the northern Baltic Proper; Boström et al. 2014) but, generally, other types of rooted vegetation dominate in the northern coastal areas. See Table S3 for further information about the uncertainty in all input data layer.

### Defining the coastal area

In this study, we have chosen to delimit the coastal area on land to areas directly adjacent to the sea with an elevation of 2 m or less, following the definition of coastal marshes by Vehmaa et al. (2024). The elevation was based on digital elevation models derived from Lidar data with an altitude resolution of 0.1 m (the models are freely available from The Swedish Mapping, Cadastral and Land Registration Authority). Some areas within the 2 m elevation limit definition did not meet the criteria for mapping coastal BC habitats. For example, lakes near the coast were excluded because they are often fringed by reed, which when found in lakes are not classified as BC habitats. Similarly, BC habitats were excluded if located within areas where man-made structures, such as roads, acted as barriers between the habitat area and the sea. The submerged coastal area was defined using the feasibility layer utilised by Huber et al. (2022) and Thomasdotter et al. (2024). This layer represents coastal areas where it, based on water quality and the quality of the satellite images, was possible to retrieve satellite-derived bathymetry information (see more details in Huber et al. (2022) and Thomasdotter et al. (2024)).

### Quality assessment and quality control

In the SAV data layer used in this study, the area of vegetation has been mapped during a 5-year period and the areal cover for 1 to a maximum of 5 years is presented in the data layer. To ensure accuracy in the produced map of the SAV distribution, we conducted an independent quality assessment to determine the optimal number of analysed years (i.e. 1–5 years) to be included in further analyses (following a similar data quality evaluation methodology as applied by Thomasdotter et al. (2024)). Our quality check mapped seagrass areas from field surveys and groundtruthing in the Gullmar Fjord in Bohuslän on the Swedish west coast (Baden et al. 2003; Gullström et al. unpublished), monitored seagrass meadows, and field data from transects and SCUBA following the national method for monitoring shallow-water vegetation along the Swedish east coast, in the Bråviken area at the Baltic Sea coast (Edlund and Siljeholm 2020) and SAV habitat sites from various compiled previous field sampling efforts across the Swedish coast. To validate the accuracy of the SAV data layer, these data were cross-referenced with the SAV data layer utilising the overlay analysis tool in GIS software. Additionally, monitoring data for the maximum depth distribution of seagrass areas (*n* = 225) along the Swedish west coast, based on information from drop-video and echosounder investigations during the years 2019–2023 (HaV 2023), were manually analysed and used to evaluate the potential under- and overestimations of the SAV input data. We expected that most seagrass meadows would be underestimated since deeper vegetated areas are harder to detect from satellite images (Huber et al. 2022). Finally, recent field sampling conducted between 2022 and 2024 from the study area (Gullström et al. unpublished) was used to validate the final map by linking the nearest correct habitat type in the BC habitat map (open wetland, forested wetland, seagrass or other types of rooted submerged macrophytes) with sampled field sites (*n* = 222). A sampling point was considered correctly mapped if it was 0–30 m from the habitat in the BC habitat map and within what was considered an area of accepted error (due to uncertainties both in the GPS information from the sampling sites and the uncertainty in the final map). If the point was > 30 m away, it was classified as incorrect.

### Landscape analysis and spatial overlap of blue carbon habitats and protected area

To estimate the potential pressure from agricultural and urban land use on the mapped coastal BC habitats, we applied a landscape metric index based on the distance to and total area of agricultural (extracted from NMD) and urban areas (freely available from the Swedish Mapping, Cadastral and Land Registration Authority;^1^) in Sweden’s coastal drainage basins. Here, all sub-drainage basins (as defined by SMHI;^2^) directly adjacent to the coast were classified as coastal drainage basins. The landscape metric index was based on the proximity to agricultural and urban areas to account for the potential impacts from some of the main human-induced threats to coastal vegetated habitats, such as nutrient runoff, pollution and coastal development. The landscape analysis was conducted separately for submerged and land-based habitats. This is a simplified index useful as a predictor of the exposure and vulnerability of coastal vegetated BC habitats to human impacts, focusing on these specific stressors. However, it does not account for other important factors such as water quality, eutrophication, hydrological connectivity or erosion processes.

The analysis generated three separate pressure maps based on: (1) proximity to agricultural areas, (2) proximity to urban areas, and (3) proportion of total agricultural and urban area within the coastal drainage basin. Each analysis assigned a relative pressure value (based on a scale from 0 to 4) to all BC habitats (see Table 2). By combining the individual pressure maps, we calculated a relative cumulative impact value (from 0 to 12) for each habitat, where the habitats within the range from 6 to 12 (representing 50% or more of the maximal value, thus indicating a notable presence of pressure) were considered being in areas of higher-level pressure. The pressure map for proximity to urban areas was based on buffer zones (delimited with diameters of 100 m, 400 m, 1000 m and 5000 m) created around each urban area within the coastal drainage basin. For the calculation of pressure values for proximity to agricultural areas, the nearest distance to agricultural land was calculated and used to classify and assign values for each BC habitat. The third analysis was based on the proportion (0–100%) of the total agricultural and urban areas within the different coastal drainage basins. A relative pressure value was assigned to each coastal drainage basin, and each BC habitat was then linked to a coastal drainage basin and given the same value. For habitats extending beyond the boundaries of coastal drainage basins, such as seagrass meadows or other rooted submerged macrophyte habitats, the nearest coastal drainage basin was assigned. Where a habitat spanned more than one coastal drainage basin, the average value of all relevant coastal basins was used. Only habitats within 500 m of a coastal drainage basin were included in the third analysis (see further details in the GIS flowchart in Supplementary Information).

**Table 2:** The set-up of the three separate pressure maps used in the landscape analysis to calculate a relative cumulative impact value for each blue carbon habitat. The three different pressure maps are based on: (1) proximity to agricultural areas (2) proximity to urban areas, and (3) proportion of total agricultural and urban area within the coastal drainage basin

Pressure map 1–2: distance (buffer zone/nearest distance; km^2^)	Pressure map 3: proportion (%)	Relative pressure value
100	80–100	4
400	60–80	3
1000	40–60	2
5000	20–40	1
> 5000	< 20	0

To assess the spatial overlap of BC habitats and protected areas (i.e. nature reserves and the Natura 2000 network), an overlay analysis in GIS software was performed. The areal overlap of the different BC habitats in the different subbasins was analysed (see Supplementary Information for a flow chart of the GIS analysis). The level of pressure of potential BC habitats within and outside protected areas was compared by performing a Student’s *t* test.

## Results

### The distribution and total area of potential blue carbon habitats in Sweden

**Fig. 4:**
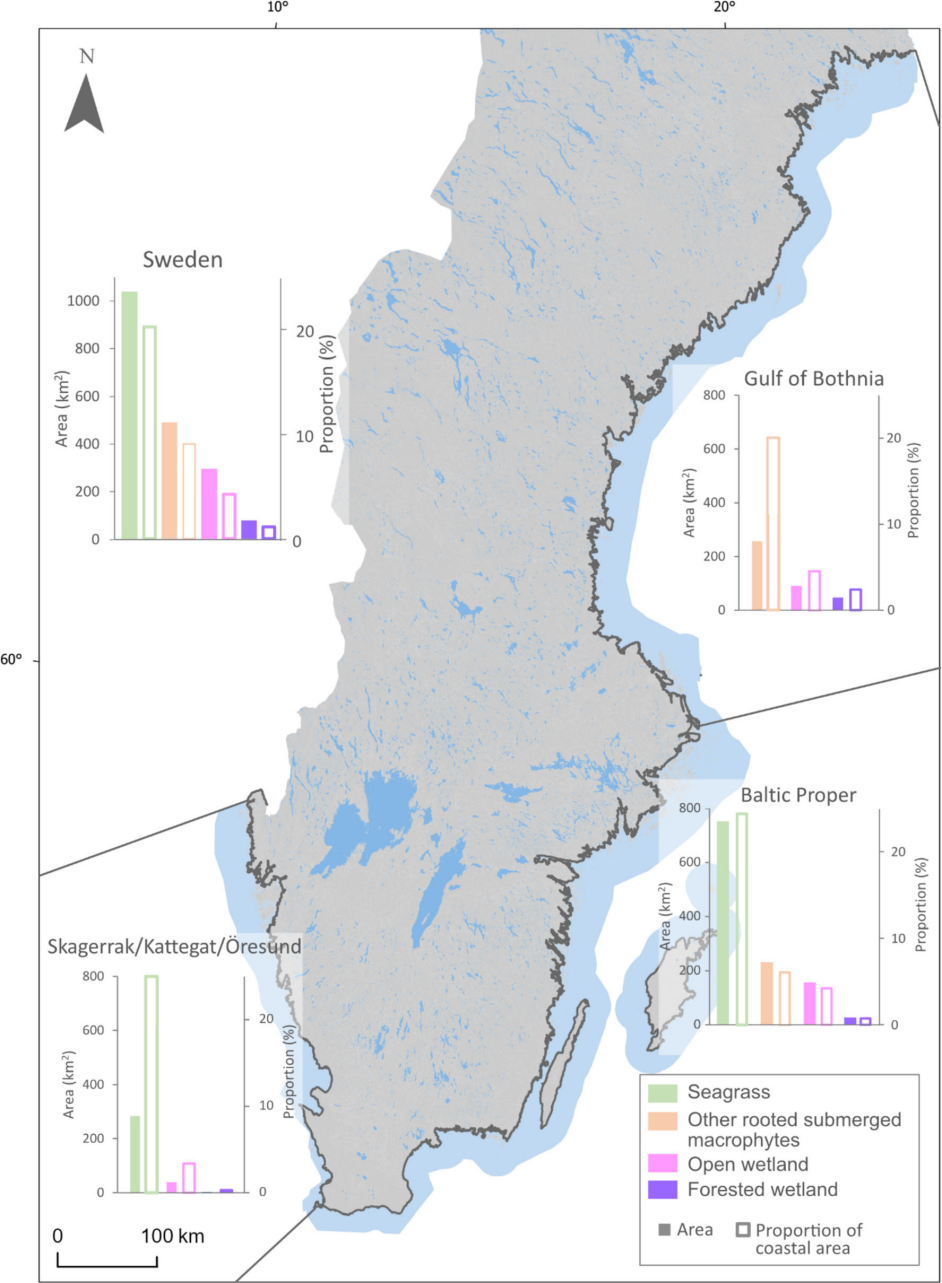
The area (solid bars) and proportion (% in relation to the delimited coastal area; open bars) of well-recognised (i.e. open wetland and seagrass meadows) and emergent (including other rooted submerged macrophytes besides seagrass and coastal forested wetlands) vegetated blue carbon habitats in all of Sweden, including the Skagerrak/Kattegat/Öresund area, the Baltic Proper and the Gulf of Bothnia

The coast, as defined in this study, covered a land area of approximately 970 km^2^. The delimited coastal area stretched from just a few meters to a maximum of around 1 km from the shoreline. The submerged coastal area covered an area of 4300 km^2^ (Thomasdotter et al. 2024), resulting in a total coastal area of around 5270 km^2^. The total area of BC habitats (well-recognised and emergent) was estimated to be around 1850 km^2^, corresponding to little over one third of the defined coastal area (Table 3). Seagrass meadows and shallow-water areas covered by other rooted submerged macrophytes were the dominating BC habitats (Fig. 4, Table 3). Seagrass meadows dominated on the Swedish west coast (i.e. Skagerrak, Kattegat and Öresund) and the southern part of the brackish Baltic Proper, while other rooted submerged macrophytes dominated in the northern Baltic Proper, Bothnian Sea and Gulf of Bothnia. Open wetlands and forested wetlands covered a smaller area compared to the other BC habitats (Fig. 4, Table 3).

**Table 3:** The area (km^2^) and proportion cover (% in relation to the delimited coastal area) of mapped blue carbon habitats in Sweden and in the different subbasins

Habitat/coastal area	Sweden	Gulf of Bothnia	Baltic proper	Skagerrak/Kattegat/Öresund
Forested wetland	58 (1%)	35 (3%)	22 (1%)	1 (0.01%)
Open wetland	267 (5%)	87 (7%)	142 (5%)	38 (4%)
Seagrass	1037 (20%)		753 (26%)	284 (29%)
Other rooted submerged macrophytes	489 (9%)	257 (20%)	232 (8%)	
Coastal area	5267	1292	2857	992
Total BC area	1851 (35%)	379 (29%)	1149 (40%)	323 (33%)

### Distance- and area-based landscape analysis

**Fig. 5:**
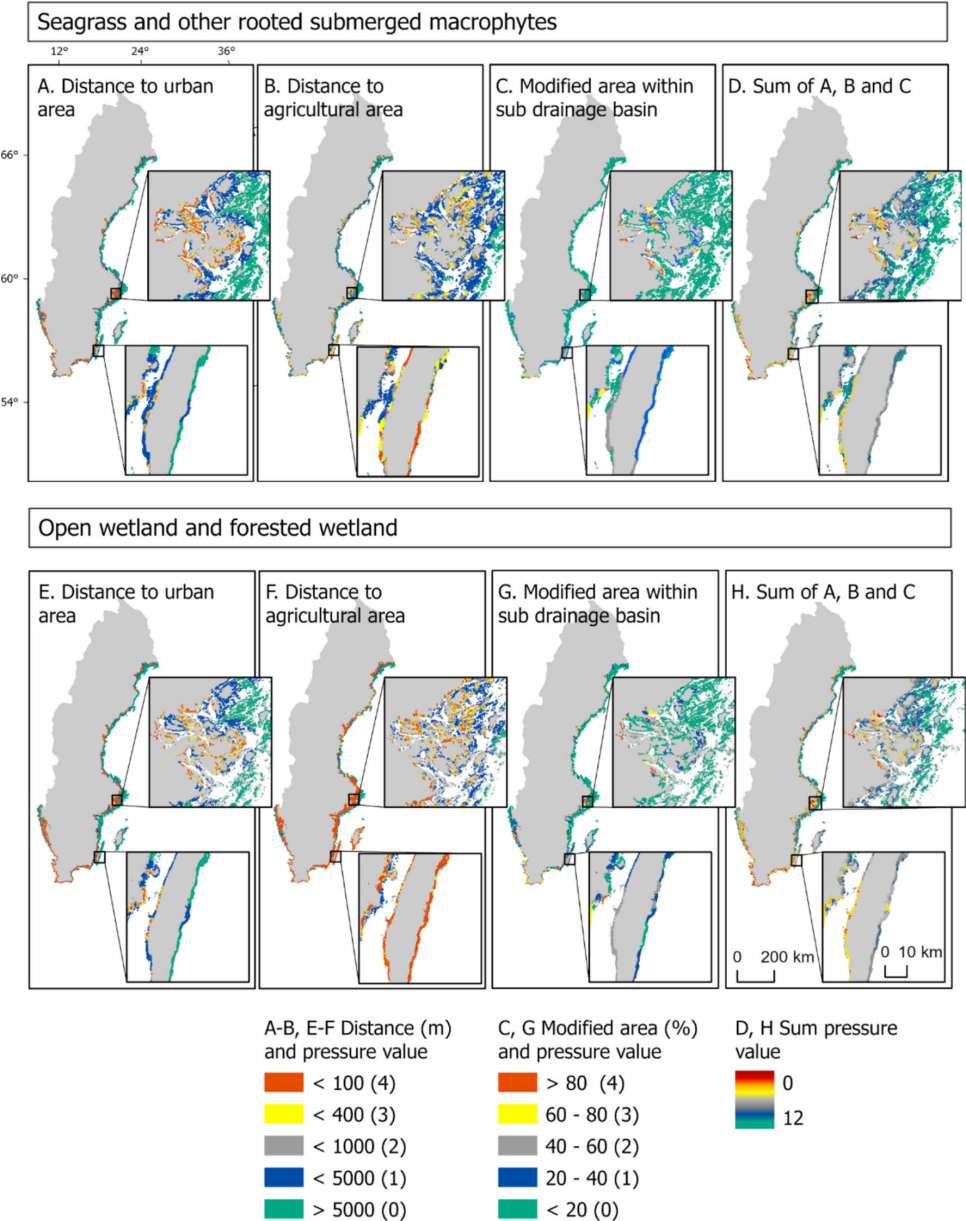
Results from the distance- and area-based landscape analysis, where the distance to and total area of agricultural and urban areas in Sweden’s coastal drainage basins were used as a proxy for the pressure of the mapped blue carbon habitats. The results are shown separately for submerged (**A**–**D**) and land habitats (**E**–**H**), and two areas, Stockholm and Öland, are zoomed in as examples. Polygons in the maps are enlarged to show general patterns

The distance- and area-based landscape analysis showed that 22% of all the mapped BC habitats were within areas of higher-level pressure (with calculated cumulative impact values of 6–12) based on their proximity to land-based human activities. The pressure levels varied among the different habitats; almost a third of the seagrass and open wetland areas were located within areas of higher-level pressure, while for other rooted submerged macrophytes and forested wetland this area was only 5% and 17%, respectively (Table 4). The pressure levels also varied between the different subbasins, where 52% of the area covered by all BC habitats in the Skagerrak/Kattegat/Öresund region were within areas of higher-level pressure, while these areas were considerably smaller in the Gulf of Bothnia (6%) and the Baltic Proper (19%). As expected, for submerged habitats, the distance- and area-based landscape analysis showed that areas closer to land were within areas of higher-level pressure compared to areas further away (Fig. 5A–D). Coastal drainage basins in the south and on the west coast of Sweden had a higher amount of modified land area compared to basins further north (Fig. 5C, G). Further, the relative importance of distance- and area-based metrics was driven by regional differences in terms of, for example, the distribution of agriculture areas (Fig. 5A, B, E, F).

**Table 4:** Area (km^2^) and areal cover [%] related to total area of blue carbon (BC) habitat of the different BC habitats within areas of higher-level pressure due to their proximity to land-based human activities and the area (km^2^) and % (areal cover) overlap of BC habitats and protected areas (nature reserves and Natura 2000 network)

Habitat/Subbasin	Area (km^2^) (and areal cover [%]) within higher-level pressure	Area (km^2^) (and areal cover [%]) overlap between BC habitats and protected areas
Forested wetland	10 (17%)	13 (22%)
Open wetland	71 (27%)	114 (43%)
Seagrass	307 (30%)	328 (32%)
Other rooted submerged macrophytes	23 (5%)	113 (23%)
All BC habitats	412 (22%)	568 (31%)
BC habitats in Gulf of Bothnia	24 (6%)	97 (26%)
BC habitats in Baltic Proper	220 (19%)	305 (27%)
BC habitats in Skagerrak/Kattegat/Öresund	168 (52%)	166 (51%)

### Spatial overlap of blue carbon habitats and protected areas

**Fig. 6:**
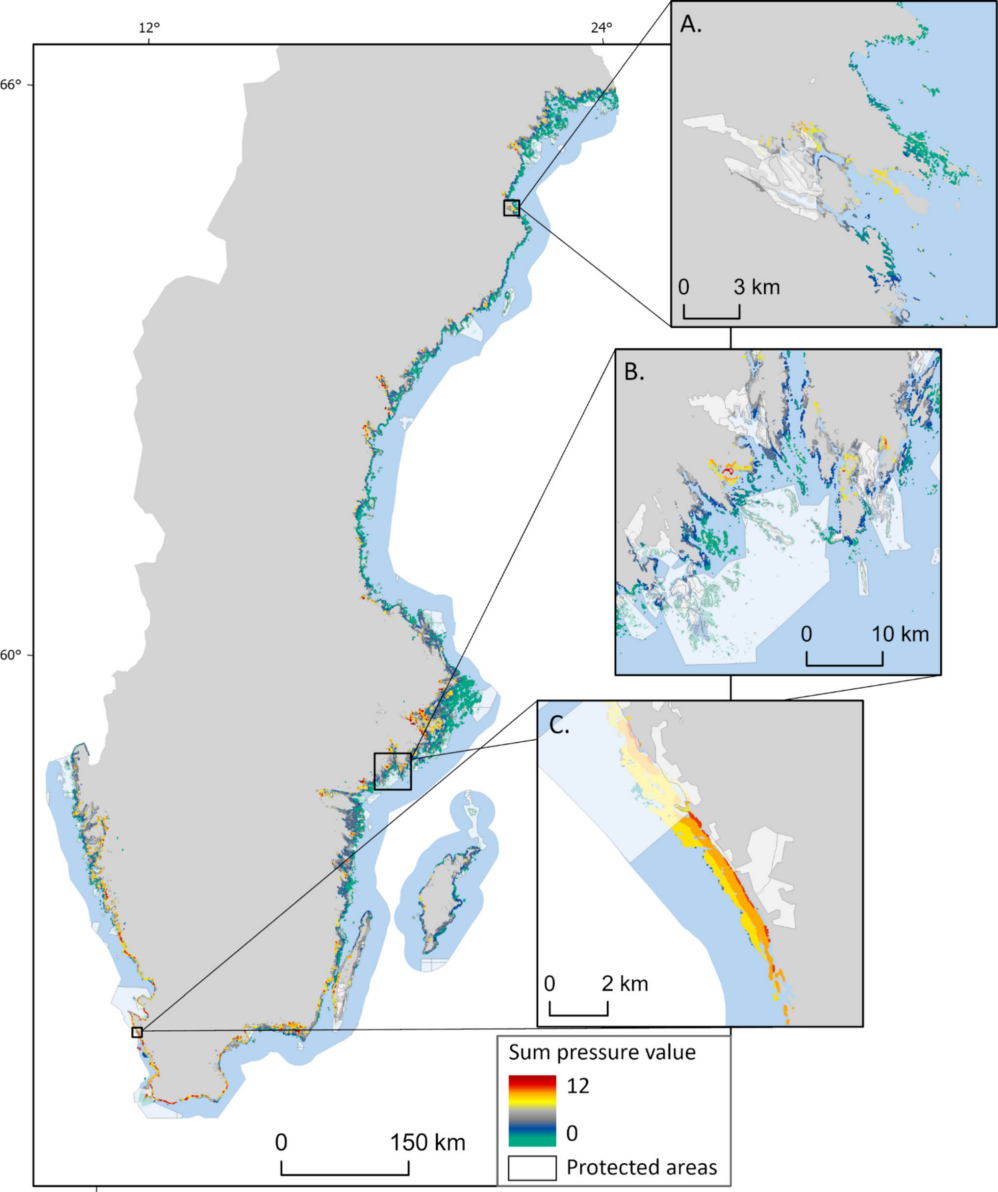
Summarised pressure values (see details in Fig. 5) for all mapped blue carbon (BC) habitats (open wetland, forested wetland, seagrass and other rooted submerged macrophytes) and distribution of protected areas (nature reserves and the Natura 2000 network) in Sweden. Well-aligned overlaps between protected areas and BC habitats (**A**), BC habitats within protected areas that, based on our landscape analysis, had lower pressure values compared to those outside protected areas (**B**), and cases where the pressure values were similar inside and outside protected areas (**C**), are shown as examples. Polygons in the maps are enlarged to more clearly show general patterns

An assessment of the spatial overlap between the mapped BC habitats and protected areas (Fig. 6) showed that 31% of the BC habitats were located within protected areas. Open wetlands had the highest overlap while seagrass, forested wetlands and other rooted submerged macrophytes had a smaller overlap (Table 4). The total area of BC habitats inside protected areas was similar in the Gulf of Bothnia and the Baltic Proper, while in the Skagerrak/Kattegat/Öresund area, over half of the mapped BC habitats were located inside protected areas (Table 4). Our landscape analysis showed that BC habitats inside protected areas had significantly lower pressure values compared to habitats outside protected areas (*df* = 24, *t* = − 2.24, *p* < 0.05), but still around 24% (corresponding to an area around 130 km^2^) of the protected BC habitats were located within areas of higher-level pressure due to their proximity to land-based human activities.

### Results from the quality control

The quality control of the produced map showed that the overall accuracy was good. When comparing the produced map with previous field sampling along the Swedish coast, almost 90% (194 of 222 sites) were correctly mapped. Submerged habitats were mapped with the lowest accuracy, but still almost 80% (27 of 34 sites), spread across the entire Swedish coastal area, were correctly mapped. For open wetlands, 91% were correctly mapped and 9% incorrectly mapped, while for forested wetlands, 78% were correctly mapped and 22% incorrectly mapped. Noteworthy, all incorrectly mapped forested wetlands were located within or in close proximity to an open wetland habitat. We concluded that a SAV coverage based on a minimum of 4 out of the 5 years was most suitable based on comparisons with field data overlap. Further, we could assess that the depth distribution was under- or overestimated in the same number of seagrass meadows (*n* = 47 and *n* = 48, respectively) and correctly estimated for 109 seagrass meadows. Out of the total 225 seagrass meadows from the depth distribution monitoring data, 21 were missing from the SAV data layer.

## Discussion

This comprehensive assemblage of coastal habitat mapping data demonstrates that over one third (around 1850 km^2^) of the extensive Swedish coast holds well-recognised and emergent vegetated BC habitats. The level of uncertainty in mapped non- or semi-submerged habitats and fully submerged habitats differed substantially. Open and forested wetlands (i.e. non- or semi-submerged habitats), which together accounted for 34% of the delimited coastal land area, were mapped with high accuracy (70–100%). Submerged habitats were estimated to cover a similar proportion (36%) of the delimited submerged coastal area, but were associated with higher uncertainty (map accuracy: 69–77%). The uncertainty for fully submerged habitats only applies to shallow sheltered areas because the mapping accuracy in deeper, more exposed areas could not be reliably quantified. Despite the challenges associated with mapping submerged habitats using remote sensing, the results highlight a large, previously unrecognised, area that could potentially comprise substantial OC stocks, while also supporting, for example, biodiversity, nutrient filtration, and coastal protection in the study area. According to the landscape-based index assessment, 22% (412 km^2^) of the coastal BC habitats were located within higher-level pressure areas, of which a quarter (around 130 km^2^) were located within protected areas. This mapping assessment of coastal BC habitats contributes to climate governance in terms of more accurate OC sink estimates and also to strategic land use and conservation prioritisation along the land-sea continuum.

### Result from the compilation of coastal blue carbon habitats

Seagrass meadows were found to be the most common BC habitat along the Swedish coastline (covering about 20% [around 1000 km^2^] of the coastal area) and compared to the estimates from previous studies (Krause-Jensen et al. 2022, and references therein), our estimates showed that seagrass covers an almost three times larger area. Thus, the seagrass distribution has likely been severely underestimated previously. Open wetlands, on the other hand, covered an area (around 270 km^2^) similar to previous estimates (Vehmaa et al. 2024). Our compilation of coastal BC habitats adds new information regarding the coverage of emergent BC habitats, i.e. other types of rooted submerged macrophytes and coastal forested wetlands, which were estimated to cover about 9% and 1% of the Swedish coastal area, respectively. The lack of relevant maps of submerged BC habitats (e.g. seagrass meadows) is a main hindrance for assessing global seagrass carbon sequestration (Macreadie et al. 2021), and therefore, regional maps contribute to more reliable OC stock estimates (Dahl et al. 2025). Coastal habitats have substantially declined during the last decades (Dunic et al. 2021; Murray et al. 2022). This mapping highlights the need for conservation and protection of current BC habitats to avoid carbon emission from eroded OC stocks following habitat decline (Krause et al. 2025). Additional emergent BC habitats, such as macroalgal beds (Pessarrodona et al. 2023) and shallow bays with a mixture of vegetated and unvegetated sediment bottoms (Wikström et al. 2025) are also common in Sweden, but were not included in this mapping. Macroalgae are, in fact, today generally considered an important BC habitat (Krause-Jensen et al. 2018; Ortega et al. 2019) and a recent study in the Baltic Proper has shown that unvegetated areas in enclosed bay systems can also be important carbon sinks (Wikström et al. 2025). Therefore, in future mapping efforts the contribution from these habitats should also be considered in OC sink assessments.

### Spatial patterns of blue carbon habitats

Due to the marine origin of *Z. marina* and given the strong salinity gradient along the Swedish coastline (Fig. 2), seagrass meadows were the most common BC habitat  in the marine environment on the Swedish west coast (including Skagerrak, Kattegat and Öresund), where they grow in sheltered to exposed areas on water depths of approximately 0.5–8 m (HaV 2023). In contrast, in the lower salinity levels (5 and lower) of the northern Baltic Proper, Bothnian Sea and Gulf of Bothnia, other rooted submerged macrophytes were the most widespread BC habitat. Generally, in the Baltic Proper, shallow vegetated habitats comprise a mix of seagrass and other types of rooted submerged macrophytes, where seagrass meadows are often found in more exposed locations on water depths of around 3–7 m (Boström et al. 2003) and other rooted submerged macrophytes often in sheltered shallow bays (Kautsky 1988; Pitkänen et al. 2013). Our assessment showed that open wetlands were equally distributed across the different subbasins, while forested wetlands were more prominent in the Gulf of Bothnia than in the other subbasins. This could be due to a general lower coastal exploitation in the northern part of Sweden. The distribution of potential BC habitats varied at regional level along the salinity gradient of the Swedish coast, where, for example, all mapped BC habitats were found in the Baltic Proper, i.e. the brackish part of the Swedish coast, playing an important role as potential sinks of OC (covering 1–26% of the coastal area). In contrast to the Baltic Proper, the extent of other rooted submerged macrophytes was limited on the Swedish west coast (due to the higher salinity level) and seagrass meadows were lacking in the Gulf of Bothnia (due to the lower salinity level). Despite these regional differences, varying types of habitats can fulfil similar BC storage functions, indicating functional redundancy in coastal OC sinks across salinity regimes. Further, the proportional area of the total amount of BC habitats varied at regional level across the coast (Fig. 2).

### Distance- and area-based landscape analysis

The results from the distance- and area-based landscape analysis (based on proximity to land-based human activities) showed clear differences between habitats and among subbasins. The habitat with the overall lowest pressure value was other rooted submerged macrophytes because a large proportion of the areal cover was found in the Gulf of Bothnia, which was the subbasin with the overall lowest pressure values. Open wetlands had the highest pressure level of all BC habitats (one third were within higher-level pressure areas). Wetlands in Sweden have been highly modified by human activities during the last century, which has led to extensive loss of wetland habitat (Naturvårdsverket 2012). Similar decreases have been observed elsewhere and generally, across the coastal areas in Europe, there has been a long-term loss of wetlands of around 50% (Beck and Airoldi 2007; Davidson 2014). A general finding from our distance- and landscape analysis is that nearshore submerged habitats, particularly those located in shallow enclosed bays, seem to encounter greater pressure than habitats located farther from land, which is consistent with the findings from a spatial risk assessment on seagrasses by Perry et al. (2020).

### Overlap between protected areas and blue carbon habitats and their pressure value

The BC habitat with the largest proportion (43% of its area) found inside protected areas was open wetlands (Table 4). The west coast subbasin (including Skagerrak, Kattegat and Öresund) showed the greatest overlap in distribution between BC habitat and protected areas, while also being the region with the largest area of BC habitats potentially experiencing higher-level pressure due to their proximity to land-based human activities. This indicates that at some  specific habitat- or subbasin levels, there is a stronger spatial correspondence between protected areas and habitats exposed to higher  anthropogenic pressure. However, when combining all BC habitats across all subbasins, habitats inside protected areas had, in fact, a significantly lower level of potential pressure from land-based activities compared to habitats outside protected areas. Although one third of all BC habitats were found inside protected areas, many habitats are reported to be in a non-favourable conservation status (Naturvårdsverket 2020). Our analysis cannot evaluate the influence of protected area on habitat status, and although a coastal area is protected, many of the threats (such as sedimentation and eutrophication) are derived from land-based activities not regulated by protected area regulations (e.g. agriculture, forestry and urban industries).

## Conclusion

This comprehensive mapping data compilation of coastal BC habitats shows that approximately one third of the long Swedish coastal zone contains both well-recognised and emergent BC habitats representing an important but previously underassessed resource in national carbon accounting, with the potential to be included in Nationally Determined Contributions. The generated spatial data provide a basis for integrated coastal management, guiding conservation priorities, land use planning and climate policy. While developed with a focus on carbon sequestration, the maps presented here also offer a foundation for assessing a broad range of other ecosystem services, such as biodiversity support, erosion control, and nutrient cycling. Further, robust, nationwide spatial data are an essential tool for achieving sustainable development in combination with effective protection of the coastal environment. Incorporating landscape-level pressure analysis further emphasises the importance of maintaining ecological connectivity to enhance the long-term effectiveness of conservation and restoration strategies.

## Supplementary Information

Below is the link to the electronic supplementary material.

Supplementary file1 (PDF 934 KB)

## Data Availability

All data are available in figures, text, tables, references and in the supplementary information. Downloadable figures (Figs. 5 and 6) available at 10.5281/zenodo.17256097.

## Footnotes

1 https://www.naturvardsverket.se/verktyg-och-tjanster/kartor-och-karttjanster/nationella-marktackedata/.

2 https://www.smhi.se/data/sok-oppna-data-i-utforskaren/huvud-och-delavrinningsomraden-svar2016.
