# Supplementary material for: Distribution of coastal blue carbon habitats in Sweden and their exposure to anthropogenic pressure
Source: Ambio. 2025 Dec 3;55(4):875–90. doi: 10.1007/s13280-025-02290-x (PMC12960885; doi:10.1007/s13280-025-02290-x)
Supplement: Supplementary file 1 — Supplementary file1 (PDF 934 KB) [file 13280_2025_2290_MOESM1_ESM.pdf]

Distribution of coastal blue carbon habitats in Sweden and their exposure to anthropogenic pressure

Supplementary Information

Table S1: Land cover and land use classes in the Swedish National Land Cover Database (in Swedish: Nationella marktäckedata, NMD) (Naturvårdsverket, 2024)

**Name**	**Definition according to the NMD product description (Naturvårdsverket, 2024)**	**Class used for mapping "open wetland" or "forested wetland"**
Forest on solid ground	Tree-covered areas on solid ground with a total crown cover of >10 % and a tree height > 5 m.	
Temporarily non-forest on solid ground	Open and re-growing clear-felled, storm-felled or burnt areas outside of wetlands. Trees are less than 5 meters.	
Forest on wetland	Tree-covered areas on wetland with a total crown cover of >10 % and a tree height > 5 m.	Used for mapping "forested wetland"
Temporarily non-forest on wetland	Open and re-growing clear-felled, storm-felled or burnt areas on wetlands. Trees are less than 5 meters.	Used for mapping "open wetland"
Open wetland	Open land where the water for a large part of the year is close by, in or just above the ground surface. Tree- and bushcovered areas are < 5 m in height except in mountain areas where it is <2 m.	Used for mapping "open wetland"
Low-growth mountain forest on wetland	Tree-covered areas on wetland in mountain areas with a total crown cover of >10 % and a tree height between 2-5 m.	
Arable land	Agricultural land used for plant cultivation or kept in such a condition that it can be used for plant cultivation. The land should be able to be used without any special preparatory action other than the use of conventional farming methods and agricultural machinery. The soil can be used for plant cultivation every year. Exceptions can be made for an individual year if special circumstances exist.	
Non-vegetated open land on solid ground	Other open land that is not wetland, arable land or exploited vegetation-free surfaces and has less than 10 % vegetation coverage	

	during the current vegetation period. The ground can be covered by moss and lichen.	
Vegetated open land on solid ground	Other open land that is not wetland, arable land or exploited vegetation-free surfaces and has more than 10 % vegetation coverage during the current vegetation period. Tree- and bushcovered areas are < 5 m in height except in mountain areas where it is <2 m.	
Low-growth mountain forest on solid ground	Tree-covered areas on solid ground in mountain areas with a total crown cover of >10 % and a tree height between 2-5 m.	
Building	A durable construction consisting of roofs or roofs and walls and which is permanently placed on the ground or partly or wholly below ground or is permanently placed in a certain place in water and is intended to be designed so that people can stay in it.	
Constructed, not building or road/railway	Artificial open and vegetation-free surfaces that are not building or road/railway.	
Road/railway	Road or railway.	
Peat extraction	Area in open wetland for extraction of peat.	
Inland water	Lakes or water-courses.	
Sea and ocean	Sea, ocean, estuaries or coastal lagoons.	

Table S2. Habitats from the “Natura habitat map” (NNK) (Naturvårdsverket, 2023) utilized to map “opem wetland” and “forested wetland”.

**Habitat**	**Habitat types**	**Natura 2000 habitat**
Open wetland	1630 - Boreal Baltic Coastal meadows	yes
Open wetland	1330 - Atlantic salt meadows	yes
Open wetland	6411 - Molinia meadows on calcareous soils	yes
Open wetland	7000, 4811 – Wetland habitat without specific classification	no
Open wetland	7999 – non-Natura 2000 wetland habitat	no
Open wetland	7140 - Transition mires and quaking bogs	yes
Open wetland	4010 - Northern Atlantic wet heaths with *Erica tetralix*	yes

Open wetland	1310 - Salicornia and other annuals colonizing mud and sand	yes
Open wetland	7210 - Calcareous fens with *Cladium mariscus* and species of the *Caricion davallianae*	yes
Open wetland	7230 - Alkaline fens	yes
Open wetland	6412 - Molinia meadows on clayey-silt-laden soils	yes
Forested wetland	9080 - Fennoscandian deciduous swamp woods	yes
Forested wetland	9925 – non-Natura 2000 forest habitat on wet soil	no

Table S3: The uncertainty for the different input data layers used for mapping Sweden's blue carbon habitats.

*These calculations mainly represent the uncertainty in sheltered shallow areas and the uncertainty in deeper and more exposed areas is still somewhat unclear

**Data layer**	**Area/habitat**	**Accuracy**	**Source**
Swedish national land cover database	Open wetland	80-100%	(Naturvårdsverket, 2024)
	Forest on wetland	70-80%	
Natura habitat map	See table 1	Different levels of mapping accuracy for different locations and region apply. Some areas are mapped based on field survey but most areas are mapped based on interpretation of aerial and/or orthophotos (which is a more uncertain method compared to field surveys).	(Naturvårdsverket, 2023)
Submerged aquatic vegetation data layer	Overall	72%*	(Thomasdotter et al., 2024)
	Bothnian Bay	69%*	
	Baltic Proper	77%*	
	Skagerrak/Kattegat /Öresund	71%*	
Modelled distribution data of benthic habitats	Södermanland	"Good prerequisite occurrence, certain prediction"<br>"very good prerequisite occurrence, certain prediction"<br>"very good prerequisite occurrence, uncertain prediction"	(Sandman et al., 2013)
	Östergötland	min 50% probability for >10% cover of *Zostera marina*	(Carlström, et al., 2010)

GIS - Flow chart

Input layers

- The Swedish National Land Cover Database (NMD) (Naturvårdsverket, 2024)
  - raster, 10 m resolution
- "Natura naturtypskartan" (NNK) (Naturvårdsverket, 2023)
  - Vector
- GSD-Elevation data, Grid 2+, © The Swedish Mapping, Cadastral and Land Registration Authority
  - Raster, 2 m resolution
- National submerged aquatic vegetation (SAV) data layer (Thomasdotter et al., 2024)
  - Vector
- Modelled distribution data of benthic habitats (NMK, "nationell marin kartering") (Carlström et al., 2010; Sandman et al., 2013)
  - Raster, 10 m resolution

Geoprocessing

- **Elevation data**
  - Elevation data was downloaded for the entire coastline of Sweden
  - Reclassified in two groups, ≤ 5 m elevation and > 5 m elevation
  - Converted from raster to polygon
  - Polygons with ≤ 5 elevation were appended to one file
  - Areas considered not part of the coastal zone (for example rivers and lakes close to the coast as well as low laying land (< 5m) further inland was manually removed by drawing polygons over their areas and then removing them by using the "erase" tool
  - The remaining area was used to define the coastal area on land
  - → layer: "coast"

- **NMD**
  - At the time of this study, two versions of the NMD exists. In the newest one (from 2023) data is missing for Gotland and part of inland northern Sweden. Since we were also interested in the coastal areas of Gotland, we used the older version (from 2018) for Gotland and the newer version for the rest of Sweden.
  - Clip NMD layer to coastal zone: The two NMD layers were limited to the coastal zone (as defined above) by using the layer "coast" and the tool "extract by mask", cell size: 10, number of bands: 1
  - The following classes from NMD were selected
  - 2018 version
    - Pine forest on wetland
    - Spruce forest on wetland
    - Mixed coniferous on wetland
    - Mixed forest on wetland
    - Deciduous forest on wetland
    - Deciduous hardwood forest on wetland
    - Deciduous forest with deciduous hardwood forest on wetland

        - Temporarily non-forest on wetland
        - Open wetland
    - 2023 version
        - Forest on wetland
        - Temporarily non-forest on wetland
        - Open wetland

- **NNK**
    - Habitats according to Table S2 were selected
    - Habitats 1330, 1310 and 1630 from NNK were intersected with "open wetland" from NMD to see how well NNK overlapped with NMD
        - NMD layer was converted to polygon (tool: "raster to polygon")
        - Overlap tested with the tool "intersect"

- **Combining NMD and NNK**
    - The different classes of forest on wetland and wetland (from the two NMD layers, which had been converted to vector, and from NNK) were combined using the tools "merge" and "dissolve"
    - → layers: "Wetland" and "Forested wetland"

- **Quality assessment SAV layer**
    - Field data was used to determine the optimal number of analysed SAV years (1 to 5 years) to include
    - The SAV file was first convert from FGDB raster to TIFF format using the tool "Copy Raster"
    - Two areas with field/monitoring data were used
        - The Gullmar fjord in Bohuslän, on the Swedish west coast
            - Mapped seagrass areas (assessed in the field) (Gullström et al., unpublished) were compared with the SAV data layer (tool: "extract by mask"). The outcome of number of SAV years (in percentage) overlapping with the mapped seagrass area was calculated.
        - The Bråviken area outside Norrköping on the Swedish east coast
            - Monitoring data (Edlund & Siljeholm, 2020) was compared with the SAV raster file using the tool "Extract values to points" and the outcome of number of SAV years (in percentage) overlapping with the mapped seagrass area was calculated.
    - → layer: "SAV 4-5 years"
    - Monitoring data for the maximum depth distribution of seagrass meadows along the Swedish west coast (HaV, 2023), was used to estimate if seagrass meadows in the SAV data layer were under- or overestimated
        - This was visually analysed by comparing the extent/distribution/ cover of seagrass meadows in the SAV data layer (utilizing all five years of mapped SAV areas) and the monitored maximum depth distribution of 225 seagrass meadows

- **Dividing the SAV data layer into seagrass and other rooted macrophytes**
    - We differentiated between SAV areas dominated by seagrass meadows and other types of rooted macrophytes based on the modelled potential distribution of *Z. marina* in Sörmland and Östergötland County (NMK) (Nyström Sandman et al., 2013).
    - NMK and SAV layer (only 4-5 year SAV coverage) was intersected using the tool "intersect"
    - From NMK, the prediction classes "good prerequisite occurrence, certain prediction", "very good prerequisite occurrence, certain prediction" and "very good prerequisite occurrence, uncertain prediction" were used for Södermanland and minimum 50% probability for >10% cover of *Zostera marina* was used for Östergötland.
    - The NMK and SAV layer does not overlap perfectly. Areas with an overlap of modelled potential distribution of *Z. marina* and SAV coverage according to the SAV data layer were classified as seagrass and areas with no overlap were classified as other types of rooted macrophytes.
    - For Counties north of Stockholm, all SAV was classified as other types of rooted macrophytes beside Seagrass and for all Counties south of Östergötland and on the west coast, all SAV was classified as seagrass.
    - → layers: "Seagrass" and "Other rooted macrophytes"

- **Producing the final map**
    - From the steps above, four data layers were created: "Wetland", "Forested wetland", "Seagrass" and "Other rooted macrophytes"
    - The area of the different layers (habitats) was calculated

- **Assessing the accuracy of the produced map based on previous field sampling (unpublished data)**
    - Done by linking the nearest correct habitat type (wetland, forested wetland, Seagrass or other types of rooted macrophytes) with sampled field sites (using the tool "spatial join", match option: closest, search within radius: 100 m)
    - A sampling point was considered correctly mapped if it was 0 – 30 m of the correct habitat. If the point was >30 m away, it was classified as incorrect (divided into two categories, 30-100 m or >100 m).

- **BC habitats inside protected areas**
    - Three layers were used for protected areas: Nature reserves from GSD-General Map © The Swedish Mapping, Cadastral and Land Registration Authority and the area of SCI (Sites of Community Importance) and SPA (Special Protection Areas) from the Natura 2000 network.
    - The nature reserve layers were intersected (using the tool "intersect") with the BC habitats, separately and combined. The area inside and outside protected areas was calculated.

- **Calculation of habitat area inside three different subbasins**

- PLC Subbasins downloaded from HELCOM map and data services
    - The overlap of the coast in HELCOM's map layers and our coast was not perfect and therefore the subbasins were manually adjusted

- **Landscape analysis**
  - The "Seagrass" and "Other rooted macrophytes" data layers were converted from raster to polygon (tool: "raster to polygon")
  - The different habitat layers ("Wetland", "Forested wetland", "Seagrass" and "Other rooted macrophytes") were dissolved using the tool: "dissolve boundaries"
  - For this analysis, habitat patches located very close to each other should be considered one habitat area, here the tool "aggregate polygons" was used and polygons with a maximal distance of 30 m were aggregated. This was only done for habitats on land since submerged habitats were not as "patchy" and therefore there was no need to aggregate.
    - Example of an area prior and post "aggregate polygons", with the distribution of wetlands mapped in pink

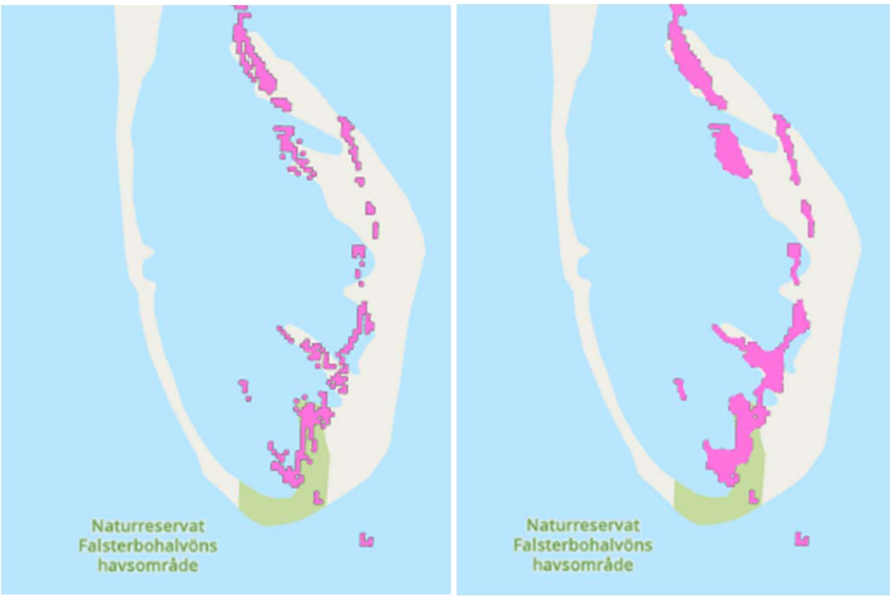

  - Selecting sub drainage basins located directly by the coast
  - Sub drainage basins were downloaded from SMHI
  - The tool "select by location" was used to select all sub drainage basins with a maximum distance of 100 m from the sea
  - All sub drainage basins smaller than 0.1 km^2 (included mainly small island) were excluded from the analysis
  - Urban areas were extracted from GSD-General Map (© The Swedish Mapping, Cadastral and Land Registration Authority
  - Agricultural areas were extracted from NMD
  - **Proximity to urban areas**
    - Buffer zones (100 m, 400 m, 1000 m, and 5000 m) were created (using the tool: "buffer") around each urban area within the coastal sub drainage basin

        - BC habitats within these zones were assigned sensitivity values ranging from 1 to 4, where sensitivity value 4 was assigned to all BC areas within the closest buffer zones (i.e. 100 m), 3 to BC habitats within 400 m buffer zones, 2 to BC habitats within 1000 m buffer zones and 1 to BC habitats in the 5000 m buffer zones (0 för habitats outside the 5000 m buffer zones).
    - **Proximity to agricultural areas**
        - The nearest distance to an agricultural area within the coastal sub drainage basins was calculated (using the tool: “Spatial join”, one to one) and used to classify and assign sensitivity values for each BC habitat. Habitats located within 100 meters of an agricultural area were given a sensitivity value of 4, those within 400 meters a value of 3, within 1000 meters a value of 2, and within 5000 meters a value of 1 (habitats further than 5000 m away were given the value 0).
    - **Proportion of total urban and agricultural area**
        - The proportion (0-100 %) of total urban and agricultural area within each coastal sub drainage basin was calculated
        - A sensitivity value of 4 was assigned to drainage basins with 80–100% modification, 3 for 60–80% modification, 2 for 40–60% modification and 1 for 20–40% modification (0 for less than 20% modification).
        - Each BC habitat was linked to a coastal drainage basin and assigned the same sensitivity value.
            - The tool Spatial join, one to one (”closest”) was used for wetlands and forested wetland
            - For seagrasses and other rooted macrophytes , which extend beyond the boundaries of the drainage basins, the closest drainage basin was assigned using the tool Spatial join, one to many (within a distance: 500 m)
            - By using the “one to many” function in “Spatial join”, some submerged habitats were “joined” to several drainage basins and in those cases the average sensitivity values for those drainage basins was assigned to the habitat
                - Example of an area were a seagrass meadow (in green) stretched along several drainage basins (marked with blue lines). Urban areas are mapped in yellow and urban areas in black.

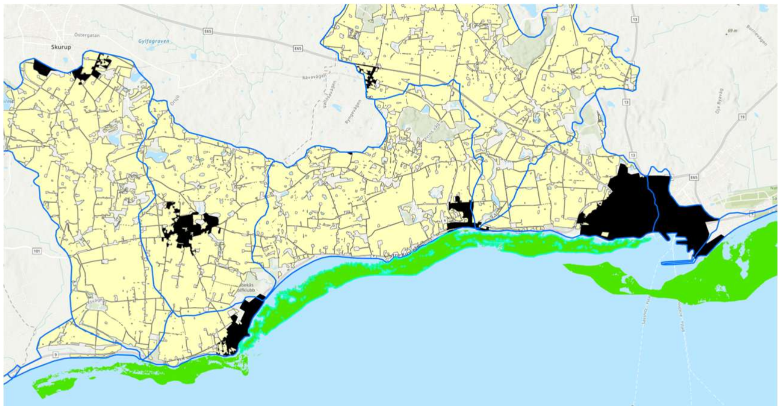

        - Since this analysis only included submerged habitats located within 500 m to a coastal drainage basin, habitats further than 500 m from land were excluded
- **Combined sensitivity**
    - The individual sensitivity maps were combined (using the tool: “raster calculator”) and a relative cumulative sensitivity value (1-12) for each habitat was calculated

References

Carlström, J., Florén, K., Isaeus, M., Nikolopoulos, A., Carlén, I., Gezelius, L., Siljeholm, E., Edlund, J., Notini, S., Hammersland, J., Lindblad, C., Wiberg, P., & Årnfelt, E. (2010). *Modellering av Östergötlands marina habitat och naturvärden*. Länsstyrelsen Östergötland.

Edlund, J., & Siljeholm, E. (2020). *Marin miljöövervakning av vegetationsklädda havsbottnar i Östergötlands skärgård 2020*.

HaV. (2023). *Vegetationsklädda bottnar Västerhavet*. Havs- och vattenmyndigheten.

Naturvårdsverket. (2023). *Naturanaturtypskartan—Beskrivning av nedladdningsbara data* (No. 1.2). Naturvårdsverket.

Naturvårdsverket. (2024). *Nationella marktäckedata 2023 Basskikt NMD2023 version 0.1* (No. 1.0). Naturvårdsverket.

Nyström Sandman, A., Didrikas, T., Enhus, C., Florén, K., Isaeus, M., Nordemar, I., Nikolopoulos, A., Sundblad, G., Svanberg, K., & Wijkmark, N. (2013). *Marin Modellering i Södermanlands län* (No. AquaBiota Report 2013:09; p. 72).

Thomasdotter, A., Berglund, J., Paz von Friesen, C., & Moksnes, P.-O. (2024). *Marin Fjärranalys Satellitbildsanalyser för nationell skattning av undervattensvegetation åren 2018-2022*.
